# The translational significance of epithelial-mesenchymal transition in head and neck cancer

**DOI:** 10.1186/s40169-014-0039-9

**Published:** 2014-11-30

**Authors:** Christian A Graves, Fadi F Abboodi, Swati Tomar, James Wells, Lucia Pirisi

**Affiliations:** Department of Pathology, Microbiology & Immunology, University of South Carolina School of Medicine, Bldg. 1 Room B43 6439 Garners Ferry Rd, Columbia, 29208 SC USA; Department of Head and Neck Surgery, Wm. Jennings Dorn VA Medical Center, Columbia, 29208 SC USA

**Keywords:** EMT head and Neck cancer, Snail, Slug, Biomarkers, TGF beta and Head and Neck cancer, EMT and HPV, Clinical trials in HNSC

## Abstract

Positive markers of epithelial-mesenchymal transition (EMT) in head and neck cancers complicate clinical management and are associated with reduced survival. We discuss recent translational discoveries in EMT and suggest additional actionable molecular pathways, biomarkers, and clinical agents.

**Electronic supplementary material:**

The online version of this article (doi:10.1186/s40169-014-0039-9) contains supplementary material, which is available to authorized users.

## Introduction

Squamous Cell Carcinoma of the head and neck (HNC) is a complex neoplastic disease that affects the face, cranium, and neck. Clinically, HNC has usually invaded vital aerodigestive tract anatomy at presentation, and often affects key sensory nerves in the peripheral nervous system. The worldwide incidence of HNC is estimated at greater than 550,000 new diagnoses per year accounting for 15–20 new cases per 100,000 individuals [1]. Despite extensive well-powered studies and the emergence of targeted therapy, 5-year survival rates in HNC remain at approximately 50% [2]. This poor survival is likely due to the fact that local invasion, lymph node involvement and metastasis are often present at the time of diagnosis. The head and neck contain nearly 40 percent of the 800 lymph nodes present throughout the body making invasive disease a serious clinical concern [3].

## Review

Local invasion and metastasis are associated with Epithelial to Mesenchymal transition (EMT) and hold negative prognostic value. Median 5-year overall survival (OS) rates in HNC have held steady at 50% but decline to 10% when local metastasis is present at diagnosis [4].

**Figure 1:**
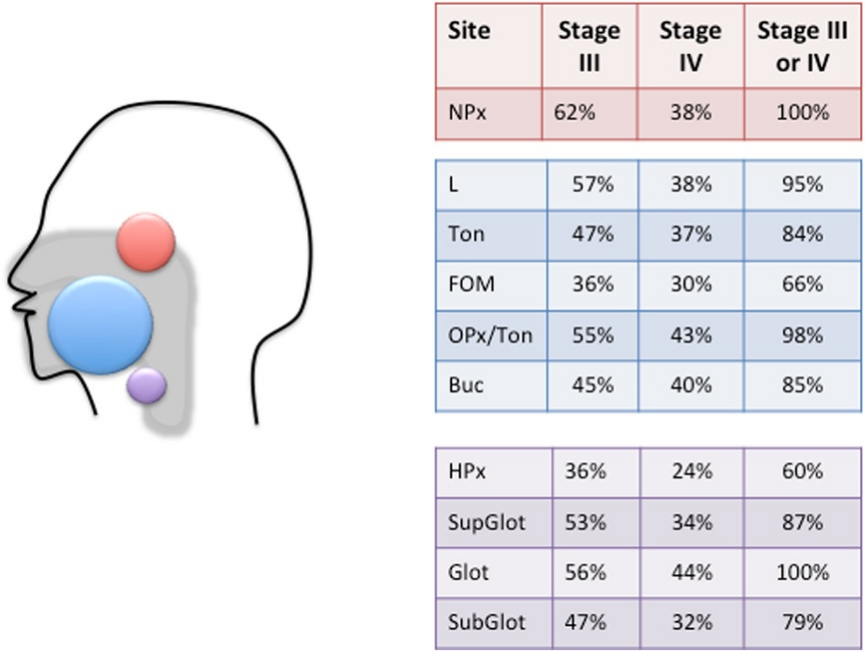
**5-year overall survival of locally invasive disease by stage and anatomic site in HNC.** Compiled from SEER data and AJCC database at cancer.org.

While differing slightly by anatomic site, local invasion in the ipsilateral field is present in ~50% of HNC at diagnosis and contralateral and bilateral invasion approach ~35% [3]. Figure 1 shows the stage at presentation of HNC by anatomic site: at all sites, HNC presents at either stage III or IV in at least 60% of the cases, and tumors of the nasopharynx, oropharynx/tonsil and glottis present at stage III or IV in over 98% of the cases. Therefore, these tumors carry a higher overall metastasis risk. Local invasion and extracapsular spread require extensive and complex resection and chemoradiotherapy [3],[5].

Genomic studies over the past decade have begun to dissect the complex underlying biology in HNC, which is often associated with tobacco and alcohol use. Furthermore, involvement of high-risk papillomavirus (HPV) as an etiologic factor in some tumors of the head and neck, first posited by Syrjänen and colleagues in 1983, has now been positively demonstrated [4],[6]-[8]. The addition of HPV-surveillance by p16 staining and molecular testing has become commonplace in the work-up of these tumors, and cases of HPV-mediated disease are on the rise [9]. Despite ample evidence for the role of HPV in cancer development at genital and skin sites in humans, it has taken several decades to connect HPV with HNC [10]. Seminal studies of canine oral papillomatosis suggested a role for EMT in tumors of the oropharynx [11],[12]. Like early observations in animals, models of papillomavirus-mediated transformation in human cells demonstrated hallmarks of EMT including induction of spindle morphology [13]. In HNC, the molecular findings of basic science studies of EMT have culminated in an ongoing effort to translate this entity into targeted therapeutics. Perhaps the greatest leaps forward have occurred in the molecular characterization of different types of HNC owing mainly to genomic, epigenomic, and transcriptomic sequencing studies [14]-[19]. In spite of this progress, EMT and the molecular underpinnings have not yet yielded robust clinical targets.

In this review, we discuss the translational relevance of EMT mechanisms in HNC and integrate these findings with the novel molecular picture emerging for this heterogeneous disease. We focus on the respective roles of viral infection and EMT in tumors of the head and neck; mechanisms of induction of EMT; the clinicopathologic outcomes associated with increased invasion; and how emerging therapies may take advantage of EMT determinants and markers.

### Translational hallmarks of EMT in embryogenesis and HNC

EMT is a highly conserved process in development, cellular physiology and wound healing in every tissue compartment. First recognized in embryogenesis, the concept of EMT as a molecular program was suggested by Hay and Greenburg in 1982 [20]. This seminal description has been extensively refined and expanded, to include epithelial loss of anchorage dependence with the basal membrane, followed by fundamental alterations in genomic and epigenomic program execution in cellular polarity and motility [21],[22]. Classic EMT-associated changes in protein expression coincide with loss of cell-cell adhesion complexes (e-Cadherin to n-Cadherin shift, increased *β-catenin* transcriptional activity) and induction of mesenchymal cell adhesion molecules *Vimentin (VIM), Fibronection (Fn).* Malignant cells also evade anoikis- or cell-microenvironment adhesion-dependent programmed cell death - by up-regulation of autocrine signalling from pro-survival factors *epidermal growth factor receptor (EGFR),* and *transforming factor - beta (TGF β)* which reduce *Bax/Bak* and *Fas/Fas-Ligand-* associated apoptosis. Loss of anchorage-dependent apico-basal orientation also corresponds with changes in cell morphology (i.e., stress *f-Actin* reorganization) and activation of GTPases (*Cdc42* and *Rac1*), activation of planar polarity transcriptional programs (*Pars/Crumbs, Snail, Slug*), and degradation of the basement membrane via proteolytic enzymes (i.e., matrix metallo-proteinases, MMPs). These alterations allow cancer cells to induce peritumoral angio- and neuro-genesis, followed by metastasis and perineural spread, respectively. A variety of well-characterized types of EMT have been described. These include Developmental (Type I); Fibrosis and wound-healing (Type II); and oncogenic (Type III) [21]. However, advances in the understanding of the contributions from various highly conserved molecular pathways have blurred the lines between these three types. The general molecular underpinnings of EMT have been extensively reviewed elsewhere [21]-[24]. Here, we focus on the parallels across EMT types and the evidence for their involvement in HNC Cancer.

During gastrulation, the embryo undergoes specific modifications, among which is the establishment of stem cell polarity. This event is particularly pronounced during the early formation of the neural epithelium of the neural crest [23]. Within the context of the head and neck, these structures undergo profound differentiation leading to the formation of sensory and craniofacial structures derived from the neuroectoderm. In craniofacial development, molecular programming revolves around signals transduced by the central EMT modulator transforming growth-factor beta (TGFβ). TGFβ-family ligands are responsible for activating a symphony of developmental transcriptional programs [24]. A growing body of evidence suggests the importance of various homeobox transcription factors, polycomb repressive complexes, and stem cell differentiation factors in this dynamic process. In cancer stem cells, EMT activation promotes aberrant activation of developmental programs downstream of canonical Wnt, Notch, and Sonic Hedgehog (SHH) pathways (Reviewed in [24],[25]).

Evidence for the involvement of these pathways in HNC development was first suggested from laser captured oropharyngeal biopsies, where increased expression of Frizzled1/3 (FRZ1/3) G protein coupled receptors (GPCRs), Dishevelled phosphoproteins (Dvl), and β-Catenin, (Wnt); Notch1/2 receptors, and Jagged ligand (Notch); and Patched (SHH) was observed [26]. *In vitro*, dysregulated Wnt/β-Catenin was shown to increase cellular invasiveness and decrease anoikis, both hallmarks of EMT [27]. Analysis of fresh frozen specimens from 22 patients showed that a reduction in inhibitory Wnt-7a with a concomitant increase in frizzled 5 (FRZ5) and Wnt-5a correlated with reduced patient survival [28]. Markers of dysregulation of intrinsic feedback responses to increased β-Catenin – such as increased expression of Dickkopf-3 (Dkk3) – have also been demonstrated to correlate with poor survival and increased metastasis in HNC patients [29]. In addition, epigenetic modifications have been detected in patient-derived cell lines demonstrating hypermethylation of Dickkopf-1 (Dkk-1), secreted frizzled-related peptides 1, 2, and 4 (SFRP1/2/4), and Wnt inhibitor factor 1 (WIF-1) [29]-[31].

### Studies of EMT in cancer-stem cells

The discovery of epithelial stem-like cell drivers in cancer has revolutionized the search for the elusive Achilles heel in many neoplasms. HNC cancer-stem like cells have been isolated from patient-derived lesions and leveraged to identify novel therapeutic targets. Mattox and Von Hoff first observed the ability of poorly differentiated patient-derived HNC cells to grow in soft agar in 1980 [32],[33]. These observations took nearly two decades to mature into the current molecular theories that – in HNC – were suggested by Braakhuis, Leemans, and Brakenhoff [34].

**Figure 2:**
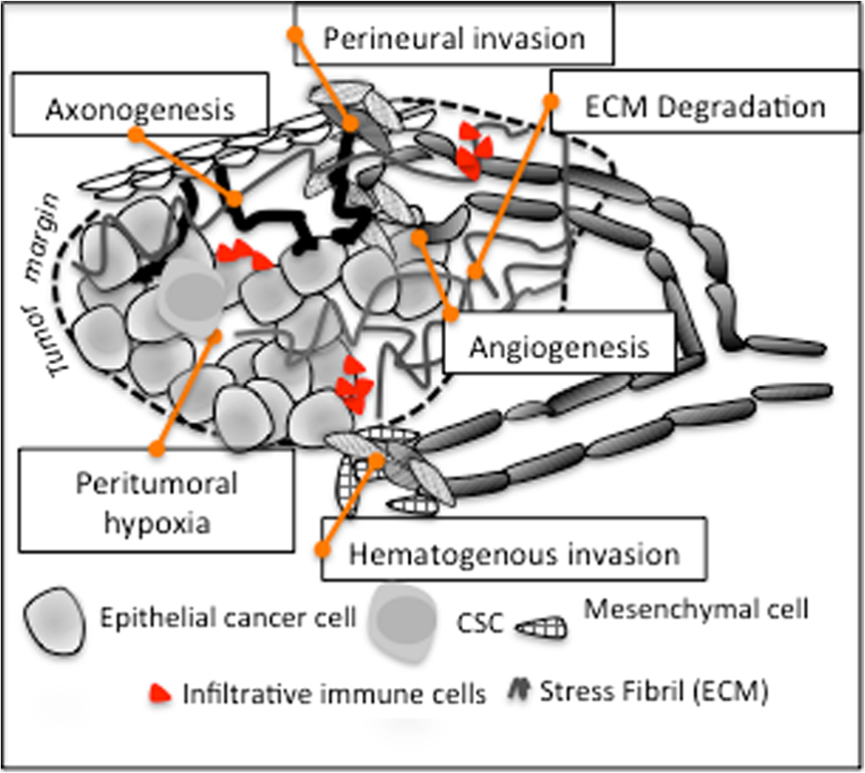
**Following genotoxic insult (Mutations (for example: loss of tumor suppressor p53, cyclin-dependent kinase N2A (CDKN2A)); HPV infection, etc.) microenvironmental signals initiate and enhance pro-EMT pathology.** Molecular signaling from the millieu promotes degradation of the extracellular matrix (ECM; Vimentin expression, matrix metalloproteinases (MMP) secretion); increased local and perineural invasion, angiogenesis, hyper mitotic features followed by hypoxia and intratumoral necrosis in parallel with tumor growth and metastasis.

A variety of stem cell molecular markers have been described in HNC including prominin-1 (CD133), the hyaluronic acid receptor CD44, Aldehyde-dehydrogenase 1 (ALDH1), and SOX2, Nanog, and OCT4, as well as basal keratinocyte marker cytokeratin 14 [35]. Importantly, CD133/OCT4/Nanog correlate with poor overall survival in a cohort of 52 oral carcinoma patients [36]. These developmental pathways synergize to drive EMT and invasiveness, are present in poorly differentiated lesions, and are linked to increased resistance to chemotherapeutic regimens. Figure 2 presents a synopsis of synergizing EMT and stem cell markers that have been linked to clinical course.

### EMT and local invasion, field cancerization, perineural invasion, and metastasis

EMT remains a controversial pathobiological model in the clinical setting, and some question the validity of the concept outside of the confines of in vitro conditions and embryonal development [37]. Despite these dissenting opinions, EMT mechanisms are still regarded as the leading hypothesis for the process of cellular loss of adhesion and spreading, and many studies, including gene expression profiling of HNC readily demonstrate the expression of markers classically associated with EMT [25],[38],[39]. At diagnosis, it is believed that 2/3 of HNC tumors harbor micrometastasis and locoregional invasion [40]. These prognostic indicators of enhanced morbidity are driven by a common feature of HNC lesions known as field cancerization. First suggested by Slaughter and colleagues, field cancerization is the development of a field of genetically altered cells in the context of a tissue. Within this altered field, the expansion of a patch of cells with increased proliferative and invasive potential gives rise to a tumor. This concept explains why cells in epithelia surrounding a tumor may present with some of the genetic and gene expression alterations found in the tumor cells [41],[42]. It is not uncommon to find micro – primary and second primaries occupying cryptic sites (such as the base of tongue or tonsillar crypts) following additional diagnostic work-up in HNC. HPV-driven HNC is clinically differentiable from HPV-negative disease due to marked reduction in field cancerization, while nodal metastases are actually more frequently present at diagnosis [43],[44].

EMT drives local invasion principally via the mechanisms discussed above and several key transcription factors governing EMT have been demonstrated in metastatic disease. Among these, the most prominent molecular features include enhanced expression of the transcription factors Snail 1 and 2 (SNAI1 and SNAI2- aka Slug) as well as SIX1, TWIST, and ZEB 1 and 2. Many of these developmental transcription factors are hijacked from their normal roles in maintaining tissue homeostasis, and their functions center around the TGF-β super family [25]. The importance of these transcription factors in head and neck development is reflected in animal studies, where mutant Snail and Slug result in craniofacial aberrations [45]. In keratinocytes, Slug plays a critical role in inducing the motile state in Type II EMT wound healing [46]. Decreases of downstream cell adhesion molecules and expression of mesenchymal markers are common features in many carcinomas and are not exclusive to HNC. Invasive HNC lesions often down-regulate E-Cadherin and up-regulate N-cadherin, vimentin, and Slug [47]. This program is increasingly detected as an early and dynamic event in the evolution of the disease. Furthermore, the E- to N-cadherin shift is sentinel in the evolution of premalignant lesions to carcinoma *in situ* and neoplasia [48] (Table 1).

**Table 1:** **EMT biomarkers currently under evaluation in the translational setting**

Marker	Prognosis	Viral-association	Biomarker type	Study power	Concomitant markers	Reference (s)
*Classic EMT pathways implicited*						
Snail 1	Poor PFS;⬆ TNM; ⬆ Metastasis; Proinflammatory	EBV^47^	IHC	N = 147 [49]	NBS; HIF1α (Hypoxia) E-/N Cadherin (Class switching)	[49]-[51]
			In vitro	N = 733 [51]		
				N = 42 [50]		
Snail 2 (Slug)	⬆ TNM; ⬆ Metastasis; ⬆ OS		IHC	N = 119 [52]	HIF1α	[52],[53]
			In vitro			
TWIST	Poor PFS; ⬆ Metastasis; ⬆ OS; ⬆Positive Nodal status		IHC	N = 147 [49]	HIF1α; Snail	[49],[54],[55]
			In vitro	N = 109 [54]		
				N = 69 [55]		
SIP1	⬆ Delayed-type neck metastasis		IHC	N = 37 [56]		[56]
			In vitro			
ZEB	--		In vitro	--		
*Canonical Developmental Pathways Implicated*						
Notch/Jagged/DLL4	--		Sequencing	N = 56 [57]	Hes/Hey1	[57]
Wnt/β-Catenin	--		IHC	N = 374 [58]		[58]
			In vitro			
Shh/GLI-1	⬆ Cetuximab resistance		In vitro			[59]
Viral EMT Pathways Implicated						
Vimentin	--	HPV	IHC	N = 69 [55]	TWIST1/2; Snail; Slug	[55]
			In vitro			
TGF-β	--	HPV	IHC	N = 140 [60]	TGFβR1/R2	[60],[61]
			In vitro	N = 200 [61]		

Note: Evidence supporting ZEB1, a key EMT transcription factor, is lacking in HNC.

In support of these observations, Slug over expression has been recently found to correlate with increased lymph node involvement and higher TNM status in a study of 119 HNC [52]. Several immunohistochemical studies have demonstrated involvement of Snail in HNC; however, these results and their impact on prognosis have been conflicting. Yang et al. showed a correlation between augmented NBS1 and TWIST and Snail expression in tissue microarrays of HNC [49],[50]. In a recent, well-designed study, 52% of cases stained intensely for Snail and this correlated with increased lymphovascular involvement, basaloid differentiation, and nodal metastasis [62]. These results are in contrast to other studies of archival specimens and related clinical data, which found no significant impact of Snail on patient prognosis [63]. The well-known oncogenic viruses Epstein-Barr Virus (EBV) has also been found to associate with enhanced EMT markers in HNC lesions [64] (Table 1).

**Figure 3:**
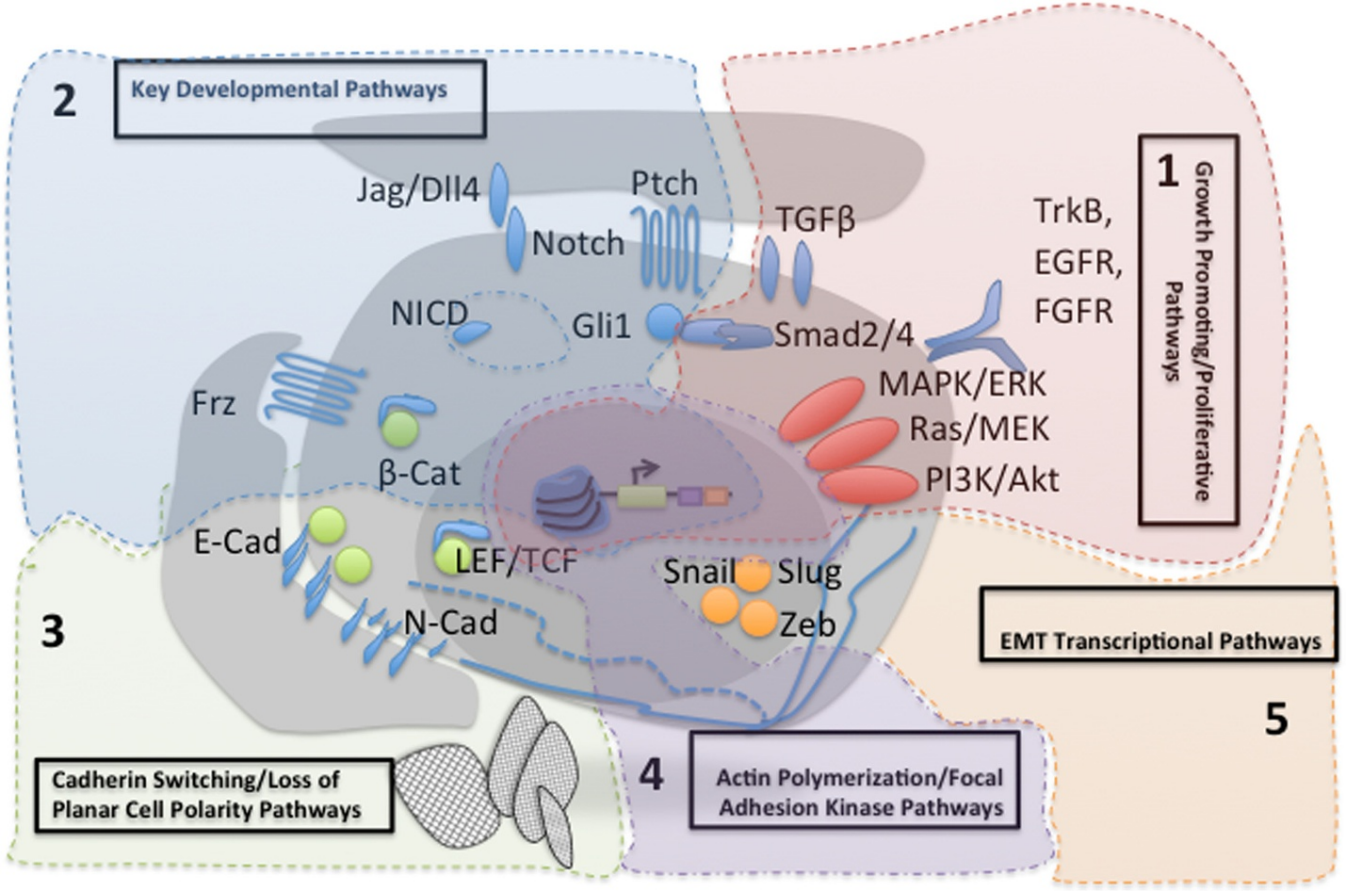
**Known molecular EMT mechanisms can be classified into 5 major overlapping categories. 1:** Growth Promoting/Proliferative Pathways are driven primarily by receptor tyrosine kinases, serine/threonine kinases, and cytokine receptors (many removed for clarity). Transforming Growth factor-beta (TGF β) and epidermal growth factor (EGFR) and other receptor tyrosine kinases (RTKs; For example: TrkB, Fibroblast growth factor receptor 1(FGFR1), etc.) signal via various intracellular kinases including mitogen activated kinase (p38/MAPK); Phosphoinositol-3-kinase (PI3k/Akt); and Ras-oncogene-MEK-ERK. TGF β signaling down-stream of TGR1 and TGR1 is multifarious and can by action of phosphorylation impact multiple kinases (via the kinase Transforming Growth Factor-β-activated Kinase (TAK1)) or SMA-mothers against decapentaplegic (Smad) transcription factors. TGF β signaling is regulated by cytoplasmic kinases (Bone Morphogenic Proteins – not discussed herein), Retinoic Acid-Retinoic Acid Receptor (RAR) and is important in developmental programs and maintaining an epithelial state. **2**: Key developmental pathways are activated by kinase activity described above or morphogens (Sonic Hedgehog (Shh)-Patched(Ptch)-Gli1; Notch-Jagged(Jag)-Delta-like Ligand (DLL1/4); or Wingless (Wnt)-Frizzled (Frz)-β-catenin (β-Cat). These canonical developmental pathways activate highly conserved transcriptional programs which center around Snail and Slug (**5**) in inducing epithelial to mesenchymal transition. Activation of Snail and Slug result in reduction of E-cadherin and diminution of adherens junctions and planar cell polarity PCP **3. 3**: Reduced and epithelial cell adhesion with concomitant increases in N-Cadherin, loss of apico-basal orientation, and reorganization of the cytoskeleton via Rho/ROCK kinases (**4**, not shown) promotes cellular motility, infiltration, and lymphadenopathy. **4**: Snail/Slug/Crumbs/Zeb signaling reduces PCP and increases cellular orientiation towards growth enhancing gradients (**1**; vasculature (angiogenesis; vasculogenesis) and nerves (axonogenesis; perinerual spreading and invasion). **5**: Feedback mechanisms and chromatin modifications by developmental pathways (**2**) perpetuate frank invasion, perineural invasion, locoregional spread, and metastasis. Not Shown: Inflammatory mediators (cytokines (Interleukins (IL) and Tumor Necrosis Factor (TNF), Chemokines (CCL and CXCR)) drive pro-inflammatory cell survival and pro-migratory pathways via hypoxia-inducible factor 1 alpha (HIF-1 α) and signal transducer and activator of transcription factor (STATs).

Interestingly, perineural invasion – which is associated with a significant decrease of overall survival and increased metastasis – is enhanced in pro-inflammatory microenvironments (i.e., elevated cytokine/NFκβ activation) and in invasive disease [51]. The mechanisms of classically defined Type III EMT and perineural spread and invasion are distinct, but have some areas of overlap. For example, a likely player in perineural invasion is the neurotrophin tropomyosin-related kinase (NTRK2 aka TrkB) which signals downstream of brain derived neurotrophic factor (BDNF) [65]. TrkB has been demonstrated to be a key regulator of EMT in HNC and TrkB overexpression leads to increased invasiveness, resulting in a refractory response to chemotherapy [66],[67]. Another line of evidence supporting the importance of neurotrophins in HNC has also emerged in the low-affinity TrkB co-receptor NGFR. Patient-based studies of NGFR- positive lesions had marked invasiveness and a locoregional recurrence rate 17 times greater than NGFR-negative lesions [68]. Fujii et al. also found a correlation between enhanced repressors of E-Cadherin and perineural invasion and demonstrated a significant association of perineural invasion with SIP1 and TWIST but not Snail expression [69]. These findings are intriguing and offer early clues on the specific extracellular interactions that promote invasiveness (Figure 3). Future translational studies will be required to dissect the relevance of these diverse mechanisms and their impact on EMT and invasion in HNC.

Closing the gap between basic science and clinical relevance will likely require more direct evidence. To this end, perhaps tumor cell tracing studies conducted with gene expression analysis of primary and metastatic tissue or evaluation of circulating tumor cells might provide some resolution to this controversy.

### HPV, EMT, and the environment: preclinical data, translational insight

HNC lesions arise from direct and indirect environmental exposure to known carcinogens including tobacco, alcohol, and oncogenic viruses. A majority of cases arise in older patients with substance-use and high-risk oral sexual practices.

In a study of 171 patients, tobacco use was significantly correlated with increased cervical metastasis (100%) compared to 54% of non-users [70]. Tobacco smoke and nicotine augment EMT in HNC models [71]. The risk-association of alcohol use and HNC is also well known. As most patients present with comorbid risks, emerging animal models are incorporating ethanol and tobacco exposure with high risk HPV infection [72].

The initial controversy surrounding the role of HPV-infection and transformation of the basal keratinocytes of the oral mucosae in HNC neoplastic transformation has largely subsided. As mentioned above, there is little clinical evidence for increased EMT in HPV-driven disease. Accordingly, gene expression profiles of HNC cancers show that markers of EMT, cell motility, invasiveness and angiogenesis predominate in HPV-negative cancers, while HPV-driven tumors are characterized primarily by alterations of the cell cycle, mitosis and proliferation [38]. However, while proliferation and cell cycle markers are virtually absent in HPV-negative cancers, HPV-positive cancers exhibit some changes related to EMT. HPV-mediated transformation of human keratinocytes *in vitro* alters TGFβ responses in such a way as to reduce the growth-inhibitory effects of TGFβ, leaving intact the EMT responses to this cytokine, therefore effectively promoting EMT. Based on these observations and on the results of gene expression profiles of HNC, we postulate that the pronounced EMT observed in HPV-negative cancers may be the result of mutations or permanent epigenetic changes, while in HPV-driven cancers EMT may be a dynamic response to TGFβ and/or to the overexpression of the homeobox transcription factor SIX1 [39],[73]-[76]. Here below we discuss some of the specific observations upon which we base this hypothesis.

**Figure 4:**
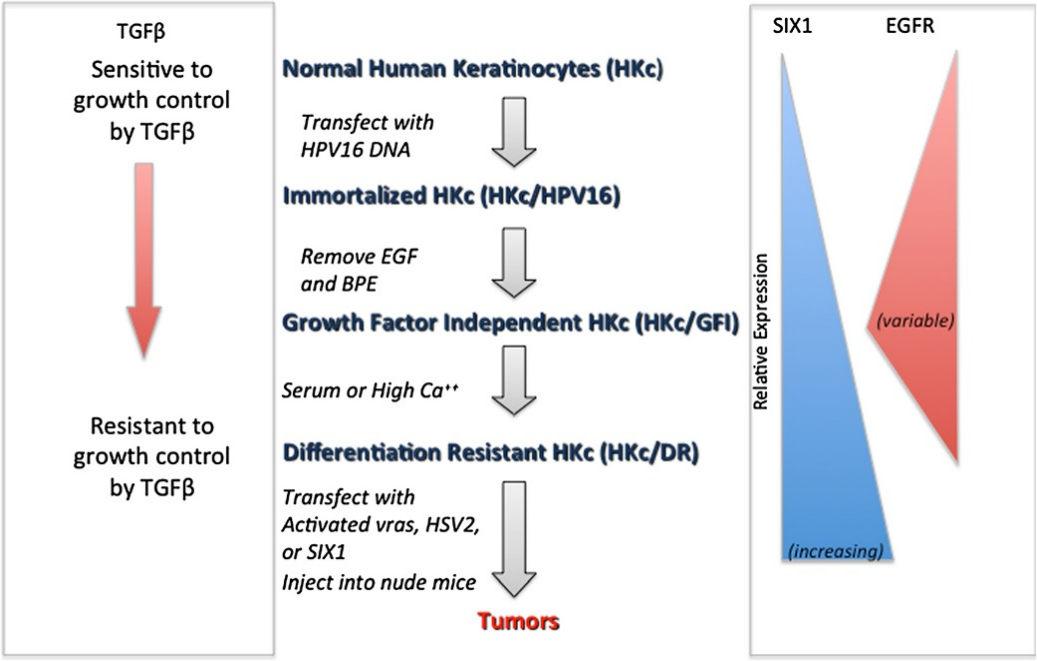
**Emerging molecular picture from >25 years of in vitro studies.** Our model of keratinocyte transformation following HPV-infection, transformation, and immortalization supports intrinsic and incremental EMT changes that center around changes in TGFβ, SIX1, and EGF.

Highly reproducible *in vitro* transformation studies in keratinocytes of various origins have provided important mechanistic insight with regards to the involvement of HPV in the transformation of human cells. We were the first to transform human foreskin keratinocytes derived from neonate donors using the HPV16 genome [77]. In the resulting model system for HPV-mediated multistep transformation of human keratinocytes (Figure 4), we demonstrated that HPV16 induces pre-neoplastic alterations that parallel many of the molecular changes detected in cervical cancer [78], therefore validating the model system as a suitable context in which to study molecular mechanisms of HPV-mediated immortalization and tumor progression. Our early studies demonstrated decreased sensitivity to the growth inhibitory effects of TGFβ, clearly attributable to a decrease in the expression of the TGFβ receptor type I [39],[73],[79]-[81] during *in vitro* progression of HPV16-transformed human keratinocytes toward a pre-malignant, differentiation-resistant phenotype (Figure 4).

We and others determined that HPV16 E7 (and presumably the E7 of other oncogenic HPV types) and TGFβ exist in a dynamic balance in HPV-transformed cells: on one side, E7 interferes with TGFβ signaling by a variety of mechanisms, decreasing overall TGFβ sensitivity and contributing to a switch from Smad-mediated (growth inhibitory in human keratinocytes) to non-canonical modes of TGFβ signaling which induce EMT in human keratinocytes. On the other side, TGFβ inhibits E7 expression by a mechanism involving the activity of NF1/Ski complexes on the HPV16 upstream untranslated region (URR) which regulates the expression of the early region [82],[83]. Hence, when canonical TGFβ signaling is robust, E7 levels are low; E7 levels rise during progression from HKc/HPV16 to HKc/DR and this leads to a switch in TGFβ signaling from canonical (growth inhibitory) to non-canonical (promoting EMT) [74]. More recently, we linked the switch in TGFβ signaling pathways in HPV16-transformed cells to alterations of the expression of the homeobox transcription factor SIX1, linked to invasiveness and aggressive behavior in a variety of cancers, including cervical cancer [76],[78]. SIX1 mRNA and protein levels increase during progression from HKc/HPV16 to HKc/DR [76],[78]. When overexpressed in HKc/HPV16, exogenous SIX1 induces EMT and the DR phenotype (59; Xu et al., Virology, in press). Forced overexpression of SIX1 in HKc/DR causes pronounced EMT and tumorigenicity [76]. While overexpression of SIX1 is clearly detected in a subset of cervical cancers ([78]; unpublished observations) and is linked to advanced disease when detected in any solid tumors, a role for SIX1 in HPV-driven HNC has not yet been proposed.

The changes associated with the emergence of EMT in sub-types of HNC – and specifically the role of EMT mediator TGFβ in inducing these changes – are active areas of investigation. TGFβ signaling occurs following ligand binding and heterodimerization of Type I and Type II TGFβ receptors. A diversity of feedback signaling and endogenous mediators and antagonists greatly increases the complexity of TGFβ signaling pathways. Canonical TGFβ signaling is transduced via a complex of Smad co-receptors present within the cytosol [80]. Smad2/3 are phosphorylated, recruiting Smad4 to form complexes that translocate to the nucleus where they interact with a host of co-activators and co-repressors to activate or repress the expression of target genes [80]. The switch in TGFβ responses from growth-suppressive to EMT-inducing involves a change from Smad-dependent to Smad-independent signaling [24] and appears to be a critical marker of progression in HPV-mediated HNC and EMT. In addition to SIX1 overexpression as we described above, the disabled homolog 2 (DAB2) – a recently characterized protein involved in mitogen response – might play a key role in regulating this switch in HNC [84]. Additionally, although not observed in mutational studies, loss of the tumor suppressor phosphatase and tensin homology (PTEN) appears to be a mechanism by which non-canonical TGFβ/Akt1/NFκβ activation manifests in an animal model [85]. Importantly, increased mutations of PIK3CA have also been described in HPV-driven HNC suggesting a potential role for the PI3k/Akt pathway in HNC [86]. The primary HPV-16 oncogenes (E6 and E7) have also been demonstrated to up-regulate Slug, TWIST, and ZEB1/2 with marked increases in ZEB transcription factors observed [87].

The studies summarized above provide preclinical targets, which have been considered in the translational setting. While not highly represented in genome-wide studies in HNC, genetic variants in TGFβ1 have been described in a cohort of 200 serum samples from oropharyngeal squamous cell carcinoma [88]. Interestingly, while the most frequently varied alleles C509T and G915C were not significant, a 2-fold risk for more aggressive disease was significantly associated with HPV-16 positive oropharyngeal lesions. These trends were most common in classically HPV-associated demographics including non-Hispanic Caucasian patients, younger age at diagnosis, never smokers and never drinkers. Moreover, frequent loss of chromosome 18q, which contains Smads 2–4 and TGRII, is observed in HNC and correlates with clinically observed invasiveness [89].

These studies have been successful in dissecting the role that HPV plays in induction of EMT and will likely drive further translational discovery.

### EMT predisposing mutations in HNC: emerging genomic, epigenomic, and transcriptomic evidence

Molecular evidence for specific chromosomal aberrations, mutations, and epigenomic drivers of EMT in HNC has recently come to light. In addition to specific targets, these studies lend important insight to the networks driving the complex pathology observed in the HNC patient.

#### Genomic mutations and EMT

Several studies have demonstrated indirect mutations in important pathways relevant to EMT. Perhaps the most direct evidence comes from the recent TCGA Pan-Cancer analysis. Kandoth and colleagues characterized the presence of significant mutations in EMT-related genes and discovered mutations in multiple TGFβ-related pathways (Smad4: 2%; TGFBR2: 3%; ACVR1B: 1.3%; SMAD2: 1%; ACVR2A: 0.7% of the 301 HNC tumors sampled) [90]. The study also described mutations in the histone acetyltransferase EP300 (8%) and confirmed previous reports of mutations in Notch1 (19.3%) being highest amongst HNC tumors [16],[90]. Recent evidence has connected EP300 with EMT as a key transcriptional activator of E-Cadherin and the adherens complex [91]. Notch is a key activator of EMT downstream of Snail and Slug signaling [25]. Notch also activates HIF1 to promote Snail expression in response to hypoxic stress in HNC (discussed above; Table 1) [92].

#### Epigenetics and EMT

**Figure 5:**
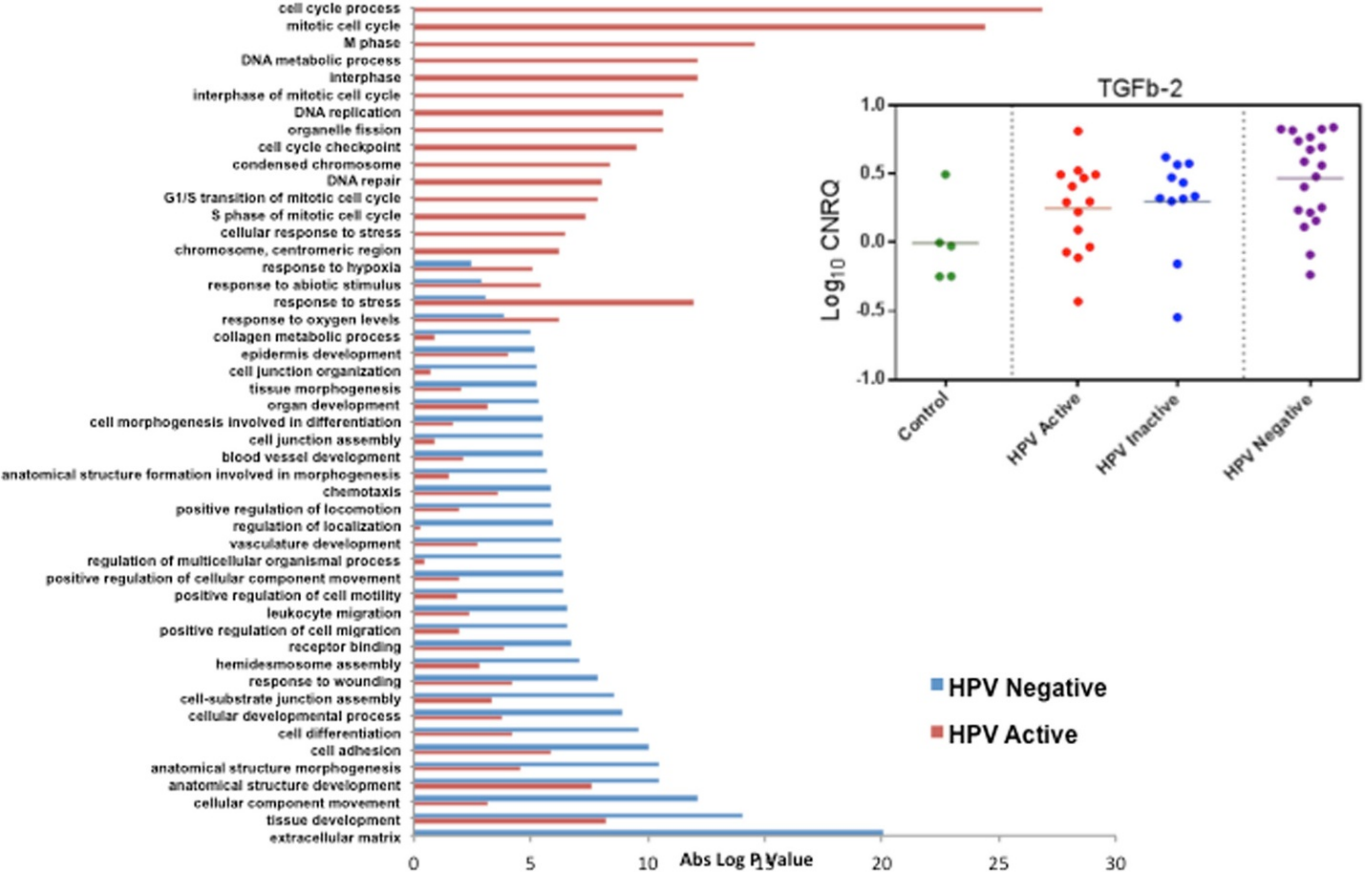
**Divergence of EMT-driven disease in HPV-positive versus HPV-negative HNC.** Gene Ontogeny (GO) pathway analysis demonstrates an increased reliance on EMT-related pro-infiltrative growth in HPV-negative disease. B: This observation is supported by an increase in TGFβ in HPV-negative (HPV^DNA-/RNA-)^versus patients with HPV^RNA+/DNA+^ lesions. *Tomar, S. et al. Submitted.*

Epigenetic modulation of key developmental and EMT-driven pathways is another area of active translational investigation in HNC. Various studies have demonstrated epigenetic down-regulation of key epithelial molecules – such as E-Cadherin. Moreover, as epigenetic sequencing technology continues to evolve, evidence has shifted to include critical roles for epigenetic regulators in the biology of HNC. Recently, it was shown that disabled homolog 2 (DAB2) – a key regulator of the TGFβ-Smad signaling – is often hypermethylated resulting in aberrant TGFβ2 signaling [84]. The divergence of mutational patterns in HPV-positive lesions versus HNC-negative lesions and the presence of lesions with HPV DNA but an absence of HPV E6/E7 RNA have recently been discovered [93]. Evidence from our laboratory suggests a divergence of gene expression in these types (Tomar et al. submitted). Furthermore, consideration of the epigenetic differences in HNC has yielded discernable methylation patterns between HPV^dna+/rna+^ versus HPV^dna+/rna-^ and HPV^dna-/rna-^[17]. It is possible that these epigenetic sequelae preempt downstream mutations observed in HNC – especially when considering tobacco use. Associations between HPV-activity and specific gene expression patterns have been described in HNC tumors. In HPV^dna+/rna-^ and HPV^dna-/rna-^, the most up-regulated gene ontologies were key to EMT pathways and included TGFβ and β-Catenin Pathways [38],[94] (Figure 5).

#### Gene expression changes and EMT

Alteration of the expression of genes commonly associated with EMT have been directly observed in HNC over the past decade. First suggested by early gene expression studies, these findings are now being translated into relevant clinical associations with patient-based gene expression assays [38],[95],[96]. Walter et al. recently described a 9 gene mesenchymal subtype discovered in a validation study of 138 patients [19]. This accounted for one fifth of the entire RNA-sequenced cohort and the 9 genes that defined it were associated with EMT centering around the transcription factor TWIST, hepatocyte growth factor (HGF), and vimentin - markers of mesenchymal phenotype. While there was no statistically significant difference in survival – likely due to the small sample size per sub-type – it was suggested that these tumors might be expected to have increased locoregional metastasis.

#### MiRNAs, post-transcriptional modifications and EMT

A great deal of interest has been focused on the involvement of neutralizing noncoding RNAs since their discovery in early 1990’s [97]. Specifically, microRNAs (miRs) have attracted attention as potential therapeutic targets. It was recently suggested that the miR-200 cluster was responsible for targeted EGFR-inhibitor resistance in HNC [98]. Furthermore, a miR profile characterized by reduced miR-345 and elevated miR-1 was found to be predictive of poor metastasis-free survival in a 64 patient cohort [99]. It will be of great interest to synergize miRNA profiles with the genomic and epigenomic mutational spectrum in HNC for the identification of novel specific therapeutic targets.

Another emerging paradigm in EMT is the importance of alternative splicing in cell adhesion molecules often aberrantly expressed in invasive disease. The hyaluronic acid receptor (CD44) has gained extensive attention due to an emerging pattern of exon splicing in HNC [100]. Analysis of the dominant three isoforms CD44 v3, v6, and v10 demonstrated enhanced stage (v3 & v6), reduced overall survival (v6 & v10), and increased regional, distant, and perineural invasion, respectively [101]. Furthermore, laminin alpha variant 3 (LAMA3) was recently discovered to carry prognostic significance in HNC in a cohort of 59 patients [102]. This variant was validated in a cohort of 44 HNC specimens where dystonin (DST), a plaque cell adhesion molecule, as also discovered [103]. Another splice variant, the nuclear protein 63 delta variant alpha (ΔNP63α) will also be of great translational interest given the involvement in epithelial stem cell biology and EMT [104],[105].

Taken together, incorporation of tissue driven molecular analysis of the sub-type specific drivers and mutational spectrum in HNC and EMT offers great translational potential. Activating mutations in PIK3CA and NOTCH1 have emerging agents (XL147, BEZ325, and GDC-0941 (PIK3CA) and OMP-52 M51 (NOTCH1) and might be potential therapeutic targets.

### Surrogate end-points and clinically actionable biomarkers of EMT in HNC

The need for clinically actionable markers of locoregional invasion and metastasis at diagnosis and for prognostic markers of recurrence is pressing in HNC. Given the accessibility of the aerodigestive tract, saliva provides a rational matrix for assessment of these markers. Most studies to date have focused on tissue- and serum-based approaches to assess changes in EMT-related cytokines, growth factors, and genes. Classic markers of EMT (TGFβ and downstream genes Smad2/6/7 and MMP9) have been described in several studies to correlate with patient survival, locoregional invasion, and therapeutic response [106],[107]. A recent study described TGFβ as a prognostic serum biomarker in HNC [108]. Additionally, PLAU and insulin-like growth factor binding protein 7 (IGFBP7) were found to be increased 1.5- and an impressive 35-fold, respectively, by plasma ELISA in HNC patients versus healthy volunteers [109]. Other correlations with markers of invasiveness, including matrix metalloproteinase 13 (MMP13) and MMP1, have also been detected in tissue and saliva [110]. Lallemant and colleagues, using a quantitative PCR approach in saliva, validated MMP1 as a specific marker at 100% but a low specificity of 20% [111]. Incorporation of soluble CD44 (sCD44), hyaluronic acid, and interleukin-8 in addition to total protein was found to have a sensitivity ranging from 75 to 82.5% and a specificity of 69.2-82.1% [112]. Additional screening for the three dominant CD44 exon variants as well as those recently validated in LAMA3 and DST as described above might also yield biomarkers with clinical relevance [103]. Evaluated extensively in epithelial cell and stem cell biology in lung cancer, ΔNP63α is emerging as a predictive biomarker in HNC in premalignant oral preneoplastic lesions [105]. While it will be of great interest to determine the role the cancer stem-like marker CD44 and ΔNP63α play in prognosis much remains to be elucidated regarding the overlap of these markers with EMT.

Many of these attempts to derive biomarkers are supported by isolated center-driven studies that sample a small population. These studies will invariably necessitate consortium-driven biomarker trials with the most promising of these EMT surrogate endpoints to produce the most meaningful translational benefits.

### Emerging potential EMT therapeutic targets in HNC

The modern armamentarium of targeted therapies in HNC has greatly expanded with the addition of molecular-based profiling and tumor sub-typing. Currently approved molecular targeted therapy Cetuximab has added epidermal growth factor receptor (EGFR), a known EMT-inducer in HNC, to the formulary [5]. However, the incomplete response rates of this novel therapeutic confirm the complex heterogeneity of HNC. As EMT accounts for extensive morbidity and varying therapeutic responses, several plausible targets and appealing regimens have emerged.

Strategies targeting fibroblast growth factor receptor 1 (FGFR1) via the non-selective receptor tyrosine kinase inhibitor PD173074 have provided promising preclinical results [113]. This novel strategy functionally reverses the EMT and induces a reciprocal mesenchymal-epithelial transition (MET) reducing Snail and Slug down-stream of AP-1 enhancement. This approach is made more appealing when considering that fibroblast growth factor (FGF) ligands are necessary for the maintenance of EMT phenotypes [114].

Other groups have targeted the inflammatory drivers upstream of EMT induction to achieve reduction in metastatic potential. Fujii and colleagues reported use of Cyclooxygenase-2 (COX-2) inhibitors – colecoxib, NS-398 and SC-91 – to increase E-Cadherin and reduce EMT-mediators (TWIST, Snail, SIP1) [69]. Dedifferentiation strategies targeting aberrant EMT-inducing Wnt and TGFβ signaling have also been suggested in preclinical studies using patient-derived tumor stem cells. For example, and colleagues provided an interesting approach using all-trans-retinoic acid, an inhibitor of TGFβ, and demonstrated reductions in β-Catenin and tumor growth [115].

Direct targeting of TGFβ signaling has proven difficult in other malignancies, due to the complexity in targeting overlapping and conserved pathways that are so important for tissue and immunological homeostasis. Various biologics and RTKIs have entered clinical trials in solid malignancies but no specific HNC trials have commenced to date [116]. Another potential limitation of these trials in HNC comes in the form of their design as they often target late stage and recalcitrant disease. It will be of great interest to establish validated biomarker-driven trials in HNC to assess objective responses to these multivalent therapeutic targets.

Searches for synergistic drug combinations in inflammatory and cellular signaling pathways carry immense potential for therapeutic interventions. Inhibitors of NFκβ (such as bortezomib) have entered clinical trials and found to be ineffective as monotherapies; however, combinatorial approaches combining targeted anti-inflammatory small molecules with RTKIs present opportunities in personalizing pharmacology to the emerging HNC sub-types [117]. Additionally, targeting strategies which incorporate targeted RTKI’s with conventional chemoirradiation models offer promise in the preclinical pipeline.

To this end, studies from the Kupferman group have indicated an important role for BDNF-TrkB in chemoresistance to standard of care platinum regimen [67]. In preclinical studies, inhibition of TrkB by AZ64, a 4-amino pyrazolylpyrimidine, in combination with Cisplatin reduced tumor growth in cell line screening [118]. It will be of interest to see if other neurotrophin RTKIs currently available in the clinical pipeline hold potential as adjuvants or in combinatorial approaches [clinical trials.gov] of pan-receptor inhibitors in clinical trials such as PLX7486, Lestaurtinib, TSR-01, or RXDX-101 Table 2.

**Table 2:** **Currently FDA approved and preclinical therapeutic targets in the translational pipeline targeting EMT-related pathways**

Agent	Type	Target/MOA	Combinational regimen	Trial	Clinical stage	References
Cetuximab	MAb	EGFR-Selective Tyrosine Kinase Inhibitor	Radiation; Cisplatin (Metastatic)	Bonner; EXTREME	FDA Approved 2006; 2008 (Metastatic)	[5],[119]
PD173074	Small molecule	FGFR1; Nonselective Tyrosine Kinase Inhibitor	--	--	Preclinical	[113]
Colecoxib	Small molecule	COX-2 Inhibitor	--	--	Preclinical	[69]
NS-398	Small molecule	COX-2 Inhibitor	--	--	Preclinical	[69]
SC-91	Small molecule	COX-2 Inhibitor	--	--	Preclinical	[69]
Trans RA	Small molecule	RAR agonist/TGFβ Antagonist	--	--	Preclinical	[115]
AZ64	Small molecule	Trkβ; Nonselective Tyrosine Kinase Inhibitor	Cisplatin	--	Preclinical	[67]
Bivatuzumab-DM1	MAb	CD44v6-Maytansionoid Cytotoxin mertansine	--	--	Phase 1 (discontinued)	[120],[121]

Targeting of stem-like cells is another strategy that has also been explored in Phase I clinical trials. The biologic bivatuzumab-mertansine has recently been explored as an anti-stem cell agent in HNC [120]. Bivatuzumab, a CD44v6- targeting antibody conjugated with the maytansinoid cytotoxin mertansine (also known by DM1), was recently shown to be well-tolerated in a dose-escalation study of 31 patients with recurrent or metastatic HNC [121]. However, despite accurate targeting of the therapeutic to CD44, this trial was terminated due to graded dose-related dermal toxicities.

## Conclusions

EMT in Head and Neck cancers continues to attract significant translational attention. The need to synergize preclinical science with relevant pathologic staging and prognosis remains a challenge. As the basic science and molecular pathways continue to evolve, the potential promise of novel therapeutic targets and improved patient outcomes stand to be realized.

## Authors’ original submitted files for images

Below are the links to the authors’ original submitted files for images. Authors’ original file for figure 1 Authors’ original file for figure 2 Authors’ original file for figure 3 Authors’ original file for figure 4 Authors’ original file for figure 5 Authors’ original file for figure 6 Authors’ original file for figure 7 Authors’ original file for figure 8

## Abbreviations

EMT: Epithelial-mesenchymal transition
HNC: Head and Neck Squamous Cell Carcinoma
OS: Overall survival
HPV: Human Papilloma Virus
HKc: Human foreskin keratinocytes (HKc/HPV16 – HPV16 transformed
HKc/DR: Differentiation resistant)
RTKI: Receptor tyrosine kinase inhibitors
MET: Mesenchymal-epithelial transition
