# Supplementary figures and images for: The translational significance of epithelial-mesenchymal transition in head and neck cancer
Source: Clin Transl Med. 2014 Nov 30;3:39. doi: 10.1186/s40169-014-0039-9 (PMC4302251; doi:10.1186/s40169-014-0039-9)

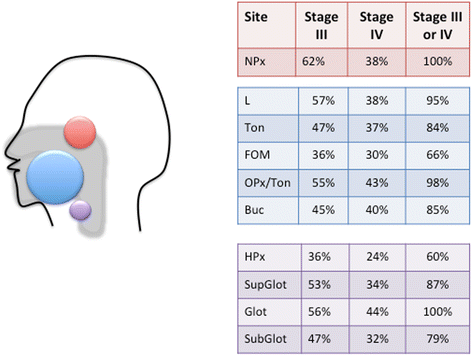

Supplement: Supplementary file 1 — Authors’ original file for figure 1 [file 40169_2014_39_MOESM1_ESM.gif]

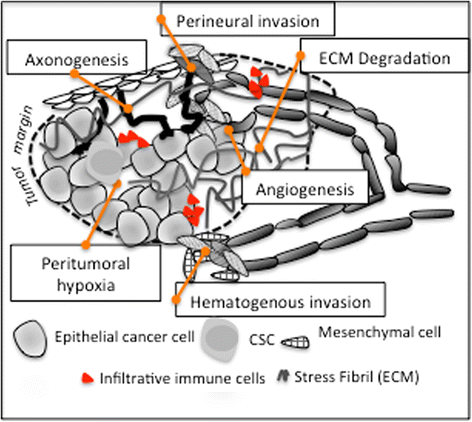

Supplement: Supplementary file 2 — Authors’ original file for figure 2 [file 40169_2014_39_MOESM2_ESM.gif]

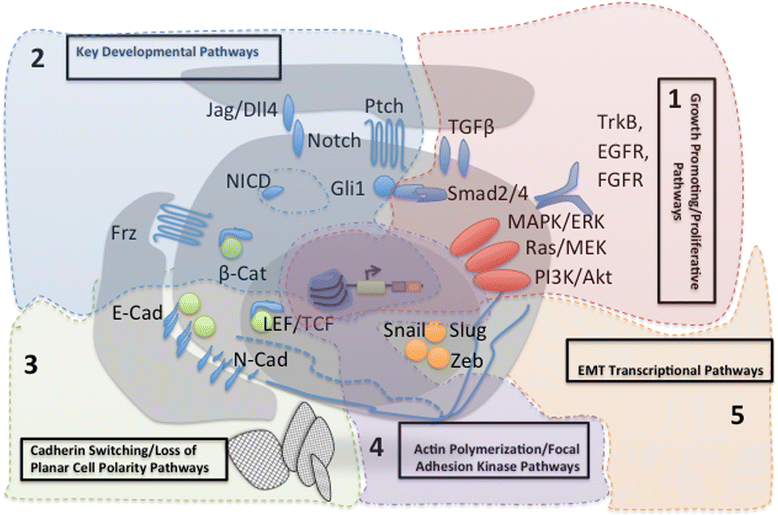

Supplement: Supplementary file 3 — Authors’ original file for figure 3 [file 40169_2014_39_MOESM3_ESM.gif]

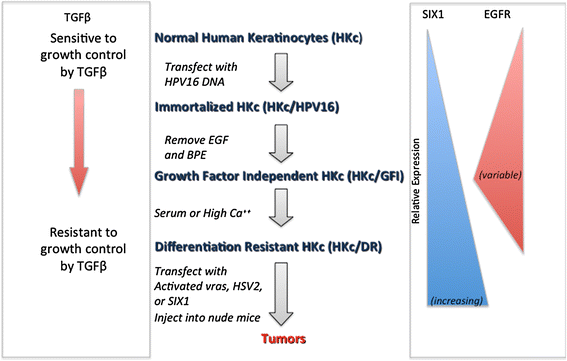

Supplement: Supplementary file 4 — Authors’ original file for figure 4 [file 40169_2014_39_MOESM4_ESM.gif]

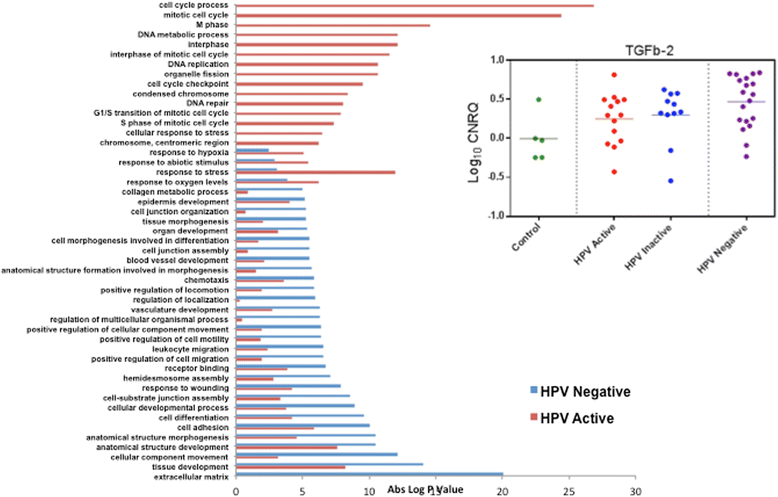

Supplement: Supplementary file 5 — Authors’ original file for figure 5 [file 40169_2014_39_MOESM5_ESM.gif]

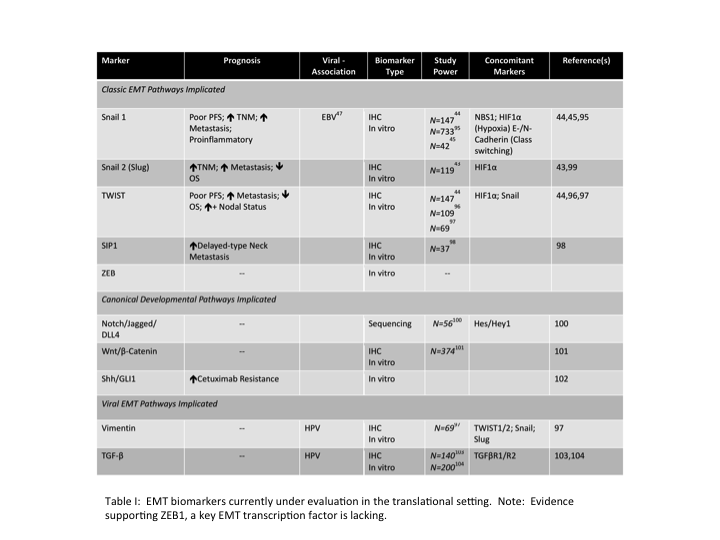

Supplement: Supplementary file 6 — Authors’ original file for figure 6 [file 40169_2014_39_MOESM6_ESM.tiff]

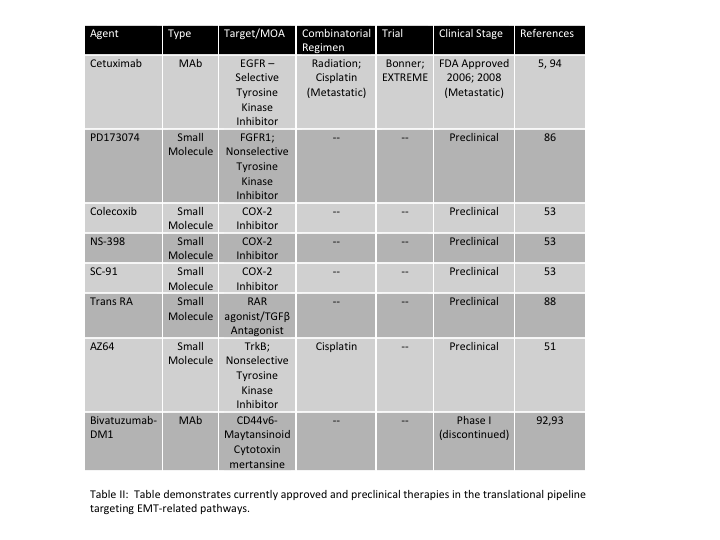

Supplement: Supplementary file 7 — Authors’ original file for figure 7 [file 40169_2014_39_MOESM7_ESM.tiff]
