# Supplementary material for: The translational significance of epithelial-mesenchymal transition in head and neck cancer
Source: Clin Transl Med. 2014 Nov 30;3:39. doi: 10.1186/s40169-014-0039-9 (PMC4302251; doi:10.1186/s40169-014-0039-9)
Supplement: Supplementary file 8 — Authors’ original file for figure 8 [file 40169_2014_39_MOESM8_ESM.pptx]

## Slide 1
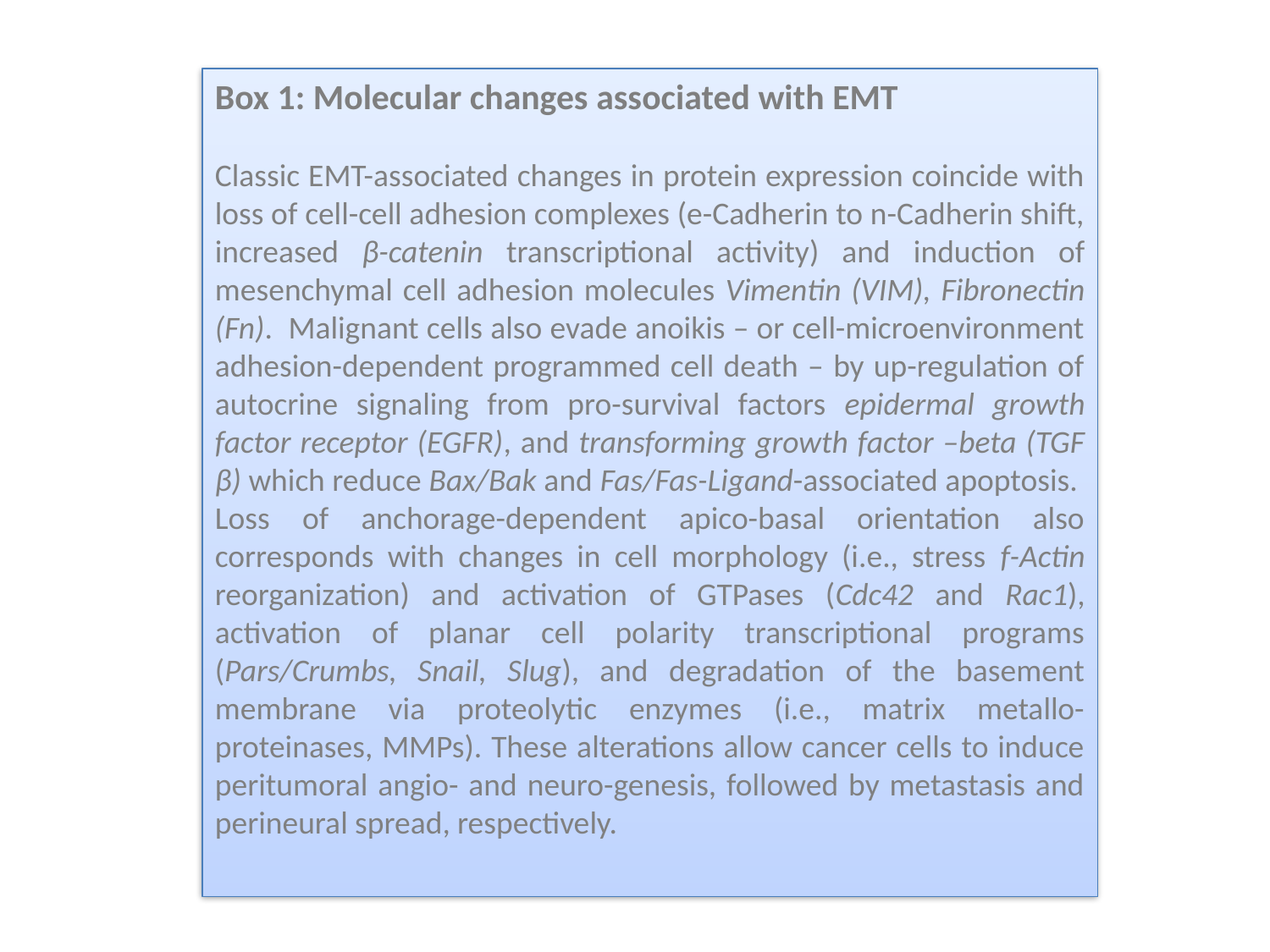

Box 1: Molecular changes associated with EMT
Classic EMT-associated changes in protein expression coincide with loss of cell-cell adhesion complexes (e-Cadherin to n-Cadherin shift, increased β-catenin transcriptional activity) and induction of mesenchymal cell adhesion molecules Vimentin (VIM), Fibronectin (Fn). Malignant cells also evade anoikis – or cell-microenvironment adhesion-dependent programmed cell death – by up-regulation of autocrine signaling from pro-survival factors epidermal growth factor receptor (EGFR), and transforming growth factor –beta (TGF β) which reduce Bax/Bak and Fas/Fas-Ligand-associated apoptosis. Loss of anchorage-dependent apico-basal orientation also corresponds with changes in cell morphology (i.e., stress f-Actin reorganization) and activation of GTPases (Cdc42 and Rac1), activation of planar cell polarity transcriptional programs (Pars/Crumbs, Snail, Slug), and degradation of the basement membrane via proteolytic enzymes (i.e., matrix metallo-proteinases, MMPs). These alterations allow cancer cells to induce peritumoral angio- and neuro-genesis, followed by metastasis and perineural spread, respectively.
